# The MYC transcription factor network: balancing metabolism, proliferation and oncogenesis

**DOI:** 10.1007/s11684-018-0650-z

**Published:** 2018-07-27

**Authors:** Patrick A. Carroll, Brian W. Freie, Haritha Mathsyaraja, Robert N. Eisenman

**Affiliations:** Division of Basic Sciences, Fred Hutchinson Cancer Research Center, 1100 Fairview Ave. N., Seattle, WA 90109, USA

**Keywords:** network, transcription, cancer, MYC, MAX, MLX

## Abstract

Transcription factor networks have evolved in order to control, coordinate, and separate, the functions of distinct network modules spatially and temporally. In this review we focus on the MYC network (also known as the MAX-MLX Network), a highly conserved super-family of related basic-helix-loop-helix-zipper (bHLHZ) proteins that functions to integrate extracellular and intracellular signals and modulate global gene expression. Importantly the MYC network has been shown to be deeply involved in a broad spectrum of human and other animal cancers. Here we summarize molecular and biological properties of the network modules with emphasis on functional interactions among network members. We suggest that these network interactions serve to modulate growth and metabolism at the transcriptional level in order to balance nutrient demand with supply, to maintain growth homeostasis, and to influence cell fate. Moreover, oncogenic activation of MYC and/or loss of a MYC antagonist, results in an imbalance in the activity of the network as a whole, leading to tumor initiation, progression and maintenance.

## Introduction: biological networks

Networks, in diverse fields including computer sciences, telecommunications, sociology, and biology, are generally defined as clusters of distinct nodes connected by what are termed “edges.” In biology, complex systems are frequently represented in terms of networks which can be subdivided into smaller groups or modules thereby revealing key relationships that underlie network activity [3,4]. The ubiquity of network organization reflects its potential to deploy interactions and functions in spatially and temporally determined patterns and to accommodate both antagonism and synergy among network components. Moreover, networks often possess “tipping points” whereby the loss- or gain-of-function of an individual network member may act to alter or distort the activity of the network as a whole. These concepts have been applied to intermediary metabolism, neural circuits, signal transduction pathways, developmental programs, immune systems, and transcriptional regulatory mechanisms. In the area of transcriptional regulation, well studied examples of networks include the genes and their encoded proteins that drive the circadian clock [6], induce pluripotency [7,8], and determine commitment to T lymphocyte differentiation [9]. In this review we focus on the MYC transcription factor network (also known as the MAX-MLX Network or the Extended MYC Network) which has been strongly implicated in normal cell growth and proliferation and in the etiology of a wide range of cancers.

### Components of the MYC network

**Fig. 1:**
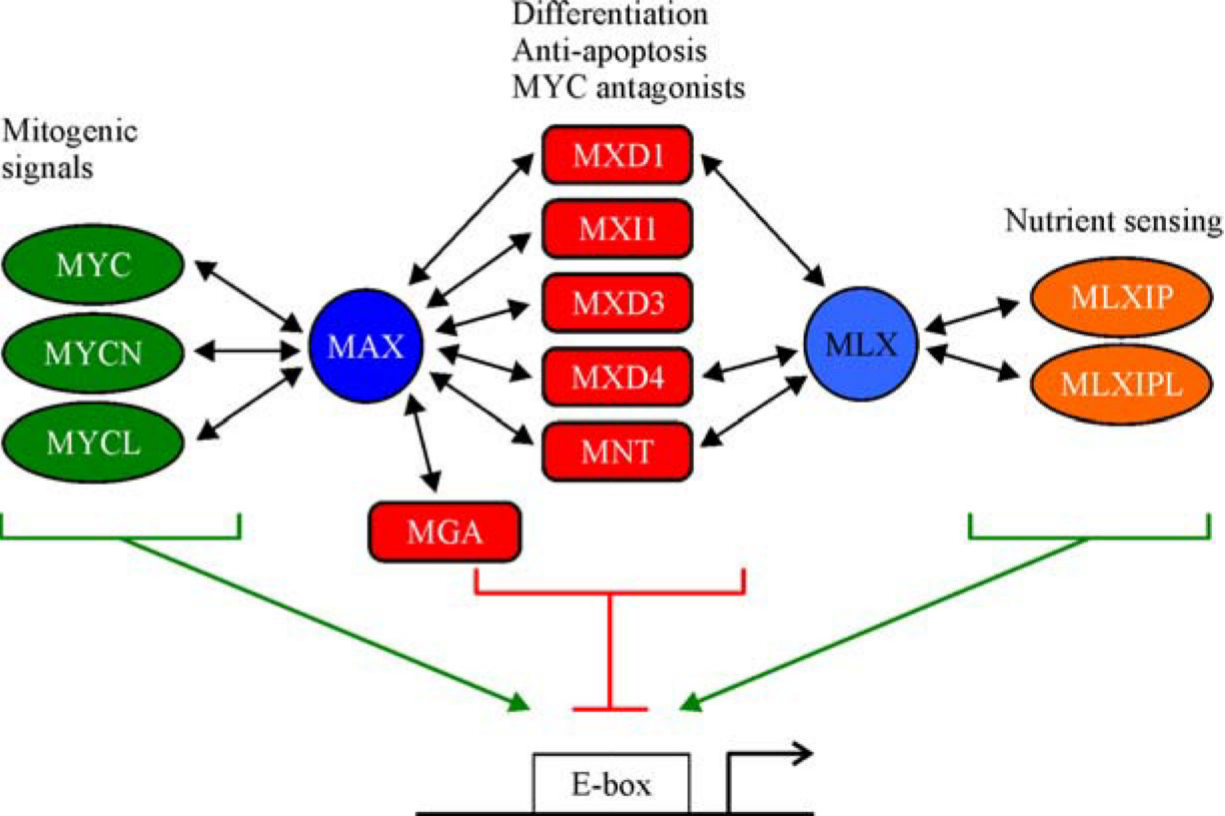
The MYC network showing the three modules from left to right—MYC; MXD/MNT/MGA; and MLXIP/MLXIPL and the dimerization interactions with MAX and/or MLX (indicated by double-headed arrows). The resulting heterodimers bind to E-box sequences in DNA.

**Fig. 2:**
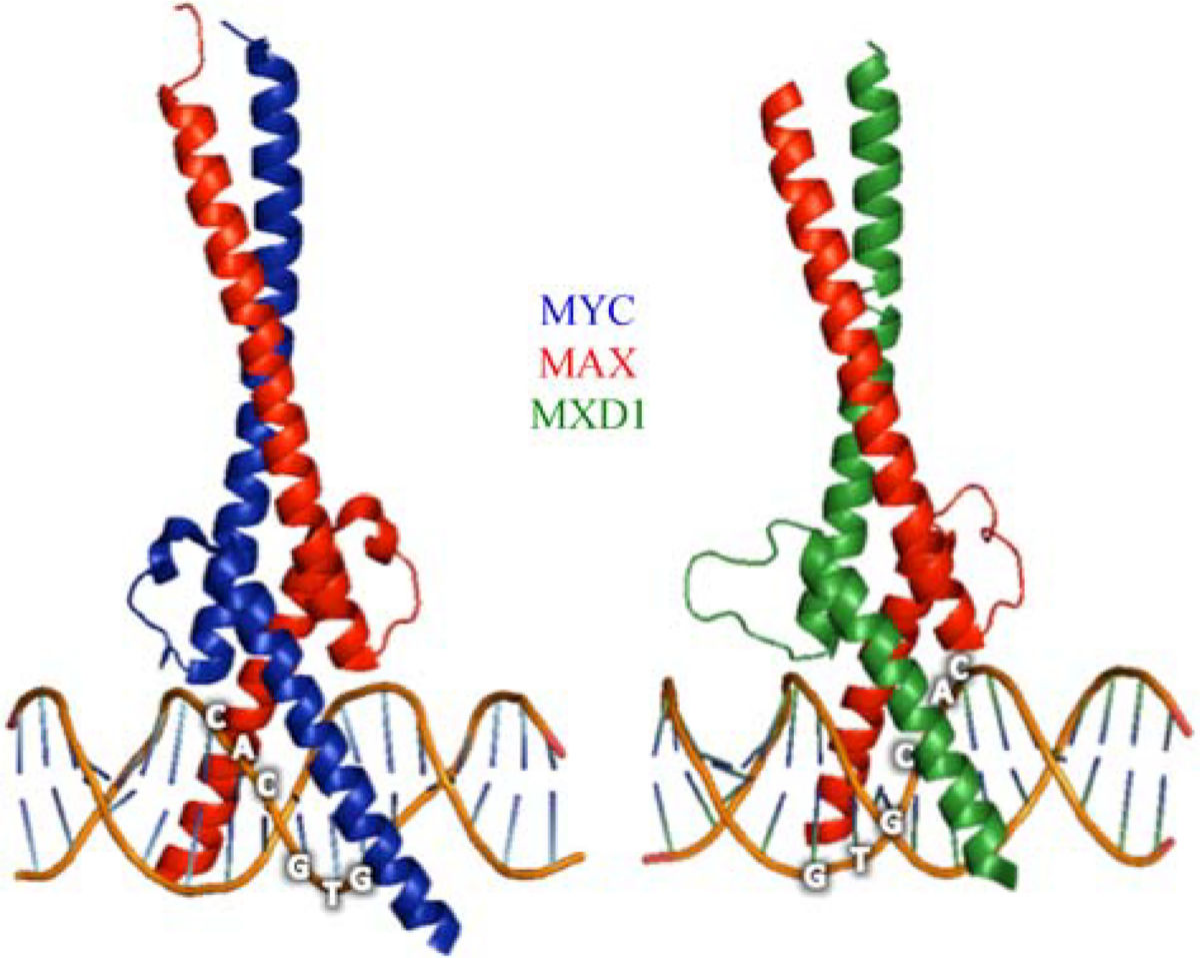
Crystal structures of the bHLHZ domains of (left) MYC-MAX heterodimer (PDB:INKP) and (right) MXD1-MAX heterodimer (PDB: INLW) bound to E-box DNA (5′-CACGTG-3′) at 19 nm and 2 nm resolution, respectively [1]. Image created with the PyMOL Molecular Graphics System, Version 1.5.0.4, Schrödinger, LLC.

Fig. 1 depicts one way to organize the components of the MYC network, all of which have the capacity to function in gene transcription and possess a highly conserved protein–protein interaction and DNA binding domain known as a basic-helix-loop-helix-zipper (bHLHZ). In its simplest sense this network can be thought of as possessing three major nodes with distinct inputs and transcriptional properties: (1) MYC family proteins; (2) proteins in the MXD family (as well as MNT and MGA); and (3) the MLXIP and MLXIPL proteins (also known as MondoA and ChREBP, respectively). Each of these network proteins employs its bHLHZ domain to form an individual heterodimer with the bHLHZ domains of either MAX, MLX or both (see Fig. 2 for crystal structures of MYC-MAX and MXD1-MAX bHLHZ domain heterodimers). It is heterodimerization with MAX and/or MLX that constitutes the functional edges of the network. In what follows we summarize the nature of the MYC network modules and focus on the functional interactions among modules (for recent reviews on the MYC network see [5,10–13]).

### MAX and MLX

Both MAX and MLX were discovered through independent protein interaction screens that sought to identify dimerization partners for MYC [14] and for MXD1 [15] respectively. The amino acid sequences of the bHLHZ domains of MAX and MLX are related to each other (~50% identity), and also show significant similarity to the bHLHZ domains of all of the MYC network proteins. Outside of their bHLHZ domains MAX and MLX do not exhibit significant homology with each other or with other network members. Importantly, the heterodimerization specificity of MAX and MLX for different network members is restricted, as indicated by the double-headed arrows in Fig. 1. MAX dimerizes with all three MYC family proteins (MYC, MYCN, MYCL) and all six of the MXD/MNT/MGA family proteins, while MLX dimerizes with only a subset of the MXD family (MXD1, MXD4, and MNT) as well as with MLXIP and MLXIPL [12,13]. Fig. 2 shows the structures of the bHLHZ heterodimer interfaces bound to E-box DNA of MYC-MAX and MXD1-MAX [1]. For proteins such as MNT, that are capable of dimerizing with either MAX or MLX, it appears that the two types of heterodimer are equivalent in terms of DNA recognition and transcriptional activity. However more detailed biophysical analysis of the binding constants related to dimerization and DNA recognition of the different complexes remain to be carried out.

Unlike MYC, MAX is capable of forming homodimers but these are inhibited from binding DNA *in vivo* due to phosphorylation by casein kinase II [16]. Furthermore structural differences in their leucine zipper regions dictate that MAX preferentially heterodimerizes with MYC or MXD rather than form MAX-MAX homodimers [1]. MLX and its several isoforms have also been reported to homodimerize and bind E-Box DNA but possess negligible transcriptional activity, as is the case for MAX homodimers [17]. Therefore, it is likely that the primary functions of MAX and MLX are to drive formation of heterodimers and facilitate their ability to specifically recognize DNA.

Both MAX and MLX are stable proteins *in vivo* with half-lives on the order of 6–8 h, while MYC, MXD, MNT, and MGA have considerably shorter half-lives, generally less than 1 h (dependent on cell type) suggesting that they are rate-limiting for heterodimer formation [18–20](H.M. unpublished data). Not surprisingly, the accumulation of these short-lived proteins is highly regulated and dependent on several factors. These include their rates of gene expression, RNA half-lives, and translation efficiencies—all processes closely linked to environmental and intra-cellular signaling. Interestingly the MLXIP and MLXIPL proteins are relatively stable compared to the MYC and MXD family proteins—possibly reflecting the fact that their regulation derives in part from their ability to shuttle between nucleus and cytoplasm (see below). The differing half-lives, localizations, and the dependence on signaling pathways are important contributors to the dynamic nature of the MYC network and its role in gene expression [10,21].

MAX serves as a network edge between MYC and the MXD/MNT/MGA family. MLX acts similarly for MXD1, MXD4 and MNT and the MLXIP/MLXIPL proteins, which comprise the third branch of the MYC Network [15,22] (see Fig. 1). Because MAX, MLX and all of the proteins in the MYC network each contain only a single bHLHZ domain, MAX and MLX do not physically connect the modules to each other. Rather, within the cellular populations of MAX and MLX proteins, individual dimerization interactions with different module members occur, based on their abundance, affinity, and localization. The heterodimers thus formed must access DNA to exert their transcriptional activities which in turn determines the overall activity of the network. In the following sections we briefly describe the properties and activities of the MYC network modules.

### The MYC module

The MYC protein family (comprised of MYC, MYCN, and MYCL) can be considered the founding members of the MYC network. The *MYC* gene was initially discovered as a oncogene (denoted v-*myc*) present in the genomes of a small group of avian retroviruses responsible for transformation of fibroblast cells in tissue culture and for different types of hematopoietic neoplasms in animals. It was subsequently determined that the v-*myc* gene was derived by retroviral acquisition of the cellular *MYC* gene [23,24]. Further studies showed that the cellular *MYC* gene (and its paralogs *MYCN* and *MYCL*), while having critical functions in normal animal growth and development, are subject to frequent alterations in a significant fraction ( > 30%) of human cancers comprising a wide range of tumor subtypes [25]. The alterations in *MYC* family genes include gene amplifications, chromosomal translocations, viral integration, and regulatory mutations in *MYC* promoter or enhancer regions. Much evidence has accumulated indicating that the alterations in *MYC* are associated with key stages of tumorigenesis including initiation, progression, and maintenance. Despite the plethora of genetic rearrangements occurring at the *MYC* locus, the vast majority of these do not directly affect the MYC protein coding region. This is consistent with the notion that it is the deregulation of MYC expression, rather than altered or neomorphic changes in its protein function, that is at the root of MYC driven cancers [26,27].

### MYC deregulation

The significance of deregulation of MYC expression in cancers became clearer when it was understood that in normal cells MYC family genes are both directly and indirectly controlled by multiple signal transduction pathways that are in turn activated by external and internal stimuli such as growth factors, mitogens, or cytokines. Many of these pathways induce MYC gene transcription as an immediate early response (i.e., not requiring protein synthesis) to mitogenic signals. For example, treatment of quiescent fibroblast cells with mitogens results in the rapid induction of MYC mRNA and protein associated with cell cycle entry [28,29]. Many mitogenic signal transduction pathways directly lead to the activation of transcription factors that engage *MYC* enhancers and promoters. Other factors regulate MYC mRNA transport, half-life and translation. Moreover, MYC protein levels are maintained by a balance between synthesis and regulated degradation. Importantly, in many cancers in which *MYC* family genes are rearranged, the tightly regulated control of MYC expression is frequently lost, resulting in constitutive expression of MYC at high levels compared to most normal cells (with the exception of normal cells during periods of high proliferative and metabolic demand, e.g., T cell activation [30]). Thus in many cancer cells MYC becomes insulated from environmental signals, the abundance of its gene products increases and it fails to be downregulated in response to appropriate signals for growth arrest and differentiation (for reviews see [10,31]). Indeed with some exceptions (e.g., where MYC loss induces a dormant state [32]) enforced downregulation of MYC in many tumors leads to regression [33,34].

### Transcriptional regulation by MYC-MAX

**Fig. 3:**
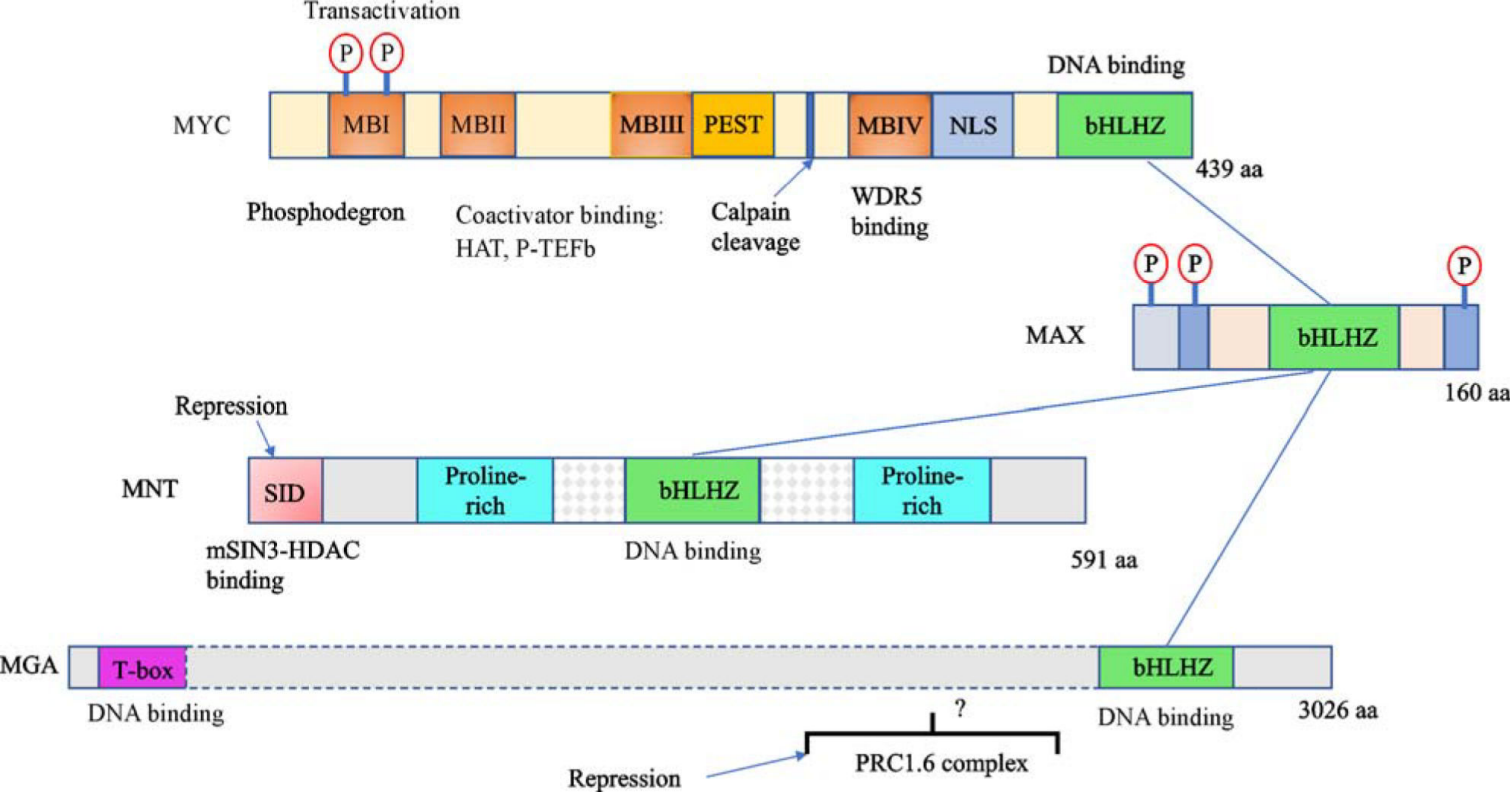
Organization of the MYC, MAX, MNT and MGA proteins. Heterodimers are formed by direct interaction of the basic-helix-loop-helix-zipper (bHLHZ) domain of MAX with the bHLH-Z domains of either MYC, MNTor MGA (blue lines). Number of residues in each protein indicated at C terminus. MYC: MBI–IV — conserved MYC boxes; PEST— region rich in proline, glutamic acid, serine and threonine; NLS— nuclear localization sequence; Calpain cleavage site—proteolytic cleavage to generate MYC-Nick [2]. MNT: SID — binding site for the mSIN3 co-repressor complex. MGA: repression mediated through assembly into a variant polycomb repressor complex (PRC1.6). Question mark indicates that the region of MGA that directly interacts with the complex is unknown. Protein lengths not to scale. See text for details.

As mentioned above, MYC family proteins function as transcriptional regulators. Heterodimerization with MAX through the HLHZ regions of both proteins permits the dimeric basic regions to form induced-fit helices that recognize the symmetric DNA sequence CACGTG (Fig. 2 and Fig. 3). This sequence, which is likely to be preferentially bound by all members of the network, belongs to the more general class of E-box sequences (CANNTG), variants of which are also recognized by MYC-MAX at lower affinity relative to the canonical CACGTG. DNA sequences harboring potential E-box binding sites are relatively frequent in the genome. For example, the canonical CACGTG sequence is present approximately every 4 kilobases. In cells, binding to genomic E-box-containing DNA is dependent on chromatin structure. The presence of histone H3 trimethylated at lysine position 4 relative to the H3 N terminus (H3-K4me3) and of other DNA binding proteins such as WDR5 have been shown to facilitate MYC-MAX binding [35,36].

While MYC-MAX heterodimers directly bind to DNA, they also recruit other proteins to genomic E-boxes. In general, these are factors that mediate transcription (Fig. 3). Several of these are associated with the N-terminal transcription activation domain of MYC, such as the NuA4 complex which contains the histone acetyltransferase GCN5, the pTEFb RNA polymerase pause-release complex, and other factors that remodel chromatin and promote transcription. Other factors, such as POZ domain transcription factor MIZ-1, bind the MYC-MAX heterodimeric interface region [37] and can significantly influence the transcriptional and biological activity of MYC-MAX [38]. The specific factors bound and their functions at genomic binding sites are likely to depend on the exact biological context [39]. Recent studies indicate that MYC-MAX binds and amplifies cell-type specific gene expression programs [40–42]. In addition, deregulated, overexpressed MYC in several tumor types binds to low-affinity non-canonical E-boxes and associates with high-density enhancers (super-enhancers) to promote expression of distinct gene subsets [40,43–47]. In other words, deregulated MYC can bind MAX and alter normal ongoing gene expression programs to impose a tumor-specific transcription profile.

In taking an overview of the MYC family branch of the network it is reasonable, as a first approximation, to view it as a protein module that integrates mitogenic signals from diverse sources and enables initiation and/or reinforcement of gene expression programs compatible with the cellular environment and the maintenance of cell fate during growth and division.

### The MXD/MNT/MGA module

In addition to forming heterodimers with MYC family proteins, MAX heterodimerizes with all members of the MXD family (MXD1, MXI1 (MXD2), MXD3, and MXD4) and the related MNT protein, each of which possesses a bHLHZ region [18,48–51] (Fig. 3). Moreover Max dimerizes with the bHLHZ domain of the MGA protein, the largest member of the MYC network, which also contains a functional T-box DNA binding region [52] related to the Brachyury/Tbx proteins, a family known to play key roles in early vertebrate development [53–55] (Fig. 3). The MAX heterodimers formed with each of these proteins bind canonical E-box DNA similar to MYC-MAX dimers and crystallographic studies indicate that the bHLHZ dimeric interfaces are nearly identical (limited to MAX-MAX; MYC-MAX, and MXD1-MAX) [1,56] (Fig. 2).

It is not entirely straightforward to ascribe a broad functionality to the MXD/MNT/MGA module as a whole. While their heterodimerization with MAX and their ability to bind E-box DNA might suggest that the MXD/MNT/MGA module functions similarly to the MYC module, a great deal of evidence argues against this idea. Indeed, MXD/MNT/MGA appear to act as antagonists of MYC function. First, whereas MYC-MAX binding predominantly promotes or reinforces active transcription (see above), the MXD/MNT/MGA module proteins act as transcriptional repressors. The MXD family members and MNT contain a short conserved amino acid sequence, which directly interacts with mSIN3A or mSIN3B co-repressor complexes [18,49,57,58]. mSIN3 acts as a platform upon which multiple proteins that mediate gene silencing are assembled. Most notably, these include Class I histone deacetylases (HDAC1 and HDAC2) that enzymatically remove acetyl groups from histones H3 and H4, thereby contributing to silencing of active chromatin [59–61] (Fig. 3). By recruiting mSIN3-HDAC co-repressor complexes to their genomic binding sites, MXD/MNT proteins appear to antagonize or reverse the transcriptional activity of MYC family proteins which, as noted above, recruit co-activator complexes leading to the acetylation of histones H3 and H4, characteristic of active chromatin (see below for discussion of antagonism).

### MGA

MGA (pronounced mega) stands somewhat apart from the MXD/MNT family due to its large size (with > 3000 residues MGA is ~14 fold larger than the MXD family and 5 fold larger than MNT) and the presence of both bHLHZ and T-box domains (Fig. 3), as well as its apparent ability to both activate or repress transcription in a context dependent manner [52]. While, like the other MXD/MNT family factors, MGA attenuates MYC-induced cell transformation it does not contain a mSIN3 binding domain. Interestingly however MGA-MAX has been shown to comprise a subunit of a variant Polycomb complex (PRC1.6) which suppresses meiosis in germ cell development and is almost certain to possess other functions [62–66]. Moreover MGA, unlike MXD family proteins, is essential for early embryonic development and has been shown to be involved in embryonic patterning and maintenance of pluripotency [67–69]. In addition, MGA sustains genomic alterations, including indels and point mutations in a range of human tumors at a high frequency relative to MXD/MNT. A subset of these alterations is predicted to inactivate the MGA bHLHZ domain proximal to its C terminus [70–74]. The prevalence of potentially inactivating mutations as well as its ability to oppose MYC transforming activity, makes MGA a strong candidate for functioning as a tumor suppressor.

### MLX and the MLXIP/ MLXIPL module

**Fig. 4:**
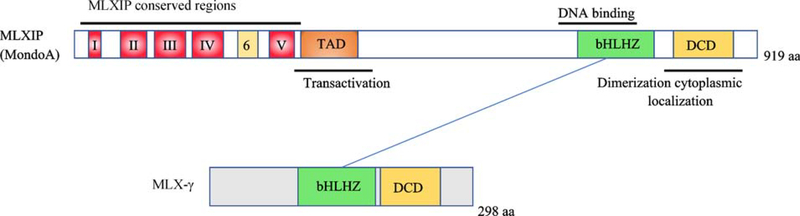
Organization of MLXIP (MondoA) and MLX. MLXIP: highly conserved regions proximal to the N terminus are thought to be responsible for binding to glucose metabolites. MLX interacts with MLXIP through their bHLHZ and DCD domains. MLX has 3 isoforms generated by alternative splicing: MLX-g (nuclear localized), MLX-α and MLX-β (both cytoplasmic).

MLX was first identified through its ability to dimerize with MXD1, MXD4 and MNT (but not to MXD2/MXI1, MXD3 or MGA) [17,22]. Moreover, MLX does not dimerize with either MAX or MYC family members. Importantly, later work revealed that MLX forms heterodimers with two large bHLHZ proteins: MLXIP (MondoA) and MLXIPL (ChREBP or MondoB) (referred to here collectively as MLXIP proteins) [22] (Fig. 4). The structural basis for the unique binding specificity of MLX is unknown but it has been suggested that a conserved tyrosine residue in the leucine zipper of the MLX bHLHZ domain may, upon phosphorylation, permit a shift in dimerization partners [11,66].

A key function of MLXIP- and MLXIPL-MLX heterodimers is to mediate the cellular transcriptional response to changes in glucose and glutamine levels. MLXIP-MLX dimers are localized to the outer mitochondrial membrane and MLXIPL-MLX dimers are present in the cytoplasm where they directly or indirectly sense G-6-P (glucose-6-phosphate) and other metabolites derived from glucose [12,75–78], relocalize to the nucleus, and bind sequences known as carbohydrate response elements (ChoREs) which are comprised of two closely spaced E-boxes [78–80]. Many of the ChoRE-containing genes bound and regulated by these MLXIP/MLXIPL-MLX heterodimers play critical roles in cellular glucose and lipid metabolism. For example, MLXIP-MLX bind to a ChoRE sequence in the promoter of the gene encoding thioredoxin-interacting protein (TXNIP) which functions in part to suppress glucose uptake and inhibit mTOR and thioredoxin (for review see [11]). In this way the MLXIP-MLX-TXNIP pathway acts as a negative feedback regulator responsive to glucose stimulation and serves as a nutrient sensor, communicating information from the mitochondrion to the nucleus in order to maintain metabolic homeostasis. The importance of this module has been underscored by studies in *Drosophila melanogaster* where MondoA-Mlx (orthologs of vertebrate MLXIP-MLX) acts as a master regulator of the physiologic response to sugar by both directly and indirectly modulating expression of effector genes mediating lipid, carbohydrate, and amino acid metabolism [81,82]. Several *Drosophila* MondoA-MLX target genes have human orthologs that contain polymorphisms associated with high levels of circulating triglycerides and coronary artery disease [81]. In addition MLXIPL-MLX regulates gene expression linked to glucose and lipid metabolism, and genetic studies further implicate this heterodimer in metabolic disease and cancer (for review see [83]).

## MYC network dynamics and crosstalk

We earlier mentioned that among the advantages of having an integrated network organization is the capacity to separate, control, and coordinate the specific activities of the network modules in time and space. This concept is likely to apply to the MYC network. Moreover, the notion of a “tipping point” — in which an imbalance in the regulation, abundance or activity of an individual network member may dysregulate the integrated function of the network as a whole — can be considered to apply to the oncogenic alterations leading to deregulation and increased expression associated with the MYC module (for review see [84]) as well as loss of function mutations in MAX [71,85] and MNT [86,87]. To understand how this might occur we will briefly describe the evidence for functional interactions among network components and argue that a functional balance among network members is critical for cellular homeostasis.

### Functional interactions between MYC and MXD/MNT

The notion that MYC-MAX and the MXD-MAX/MLX arms of the network may act antagonistically was initially prompted by the findings that MXD/MNT proteins contain transcriptional repression domains that recruit histone deacetylases to their DNA binding sites in chromatin while, in contrast, MYC proteins recruit complexes that promote transcription by facilitating chromatin accessibility and release of paused RNA polymerase (Fig. 3). The recruitment of distinct complexes with presumably opposing activities is the molecular basis for MYC vs. MXD/MNT/MGA antagonism (Fig. 3) which is also manifested at the level of their biological activities. MYC family genes, when overexpressed, stimulate cellular growth and proliferation balanced by apoptosis, eventually leading to transformation and tumor formation. By contrast, there is considerable evidence that MXD1, MXD2 (MXI1), MXD4 and MNT act to retard cellular growth and proliferation and have the capacity to block MYC induced mitogenic effectors and transformation [18,49,88–91].

One setting where this antagonism serves to regulate normal cellular events is during cell cycle entry. In the G0 to G1 transition MNT-MAX levels are constant while MYC is strongly induced, resulting in an increased ratio of MYC-MAX:MNT-MAX complexes, consistent with the idea that MYC and MNT are in competition for available MAX. Either MNT overexpression or MYC loss inhibits cell cycle entry, suggesting that antagonism between these proteins and their balanced expression may set the threshold for the transition between quiescence and proliferation [92]. However, given the multiple cellular processes that are responsive to MYC, it would seem unlikely that MXD/MNT would antagonize every aspect of MYC function. For example, there is evidence for dependence or cooperation between MYC and MNT, especially in situations in which MYC levels are elevated and MNT acts to suppress MYC-induced apoptosis [93,94]. Another example is MXD3, which has been reported to stimulate neural cell proliferation and promote apoptosis in response to radiation damage [95,96]. Although the precise relationship of MXD3 to MYC activity is unclear, it is possible that MXD3 can cooperate with or substitute for MYC.

The above examples of functional interactions within the network occur in contexts where MXD/MNT/MGA and MYC proteins are present at the same time and in the same sub-cellular compartment. However significant interactions between MYC and MXD/MNT may also occur if the proteins in question are not simultaneously present. This is relevant considering that endogenous expression of many of these proteins are correlated with different cell cycle and differentiation states. MYC expression is predominantly, but not solely, confined to proliferating cells while MXD1, MXI1, and MXD4 are detected in resting or differentiated cells and are downregulated when cells enter the cell cycle. This inverse correlation with MYC expression does not hold for MNT, which is co-expressed with MYC but is maintained upon differentiation, when MYC is downregulated (for review see [97]). However, antagonism may occur even if the MYC and MXD/MNT proteins are not simultaneously present. For example, during terminal differentiation MYC is generally downregulated while MXD1 is sharply induced as cells arrest growth. During this period a shift from MYC-MAX to MXD1-MAX complexes is observed, leading to binding and repression by MXD1-MAX at promoters previously transcriptionally activated by MYC-MAX [19,89,98]. Why would repression by MXD1-MAX at these promoters be necessary if MYC is already downregulated? One likely explanation is that loss of MYC alone is insufficient to fully suppress target gene expression. Most transcription is regulated by multiple factors and at multiple levels such as chromatin mediated promoter accessibility, pre-initiation complex assembly, RNA polymerase initiation, pausing, elongation, and termination. Recent evidence suggests that MYC, rather than acting as an transcriptional on/off switch, is primarily involved in release of paused RNA polymerase II and the amplification of gene expression [41,42]. Furthermore MYC binding leads to histone acetylation and other chromatin modifications compatible with active transcription. Therefore simply removing MYC may leave certain promoters susceptible to stimulation or induction and permit them to remain active, hence the need for expression of MXD1 to reverse MYC’s activity as an early event in differentiation. This concept is supported by genetic studies in *Drosophila melanogaster* where deletion of *dm1* (which encodes dMyc, the *Drosophila* ortholog of MYC) produces growth arrest at an early stage of larval development and diminished expression of growth related genes. By contrast, flies lacking both dMyc and dMnt (the single paralog of MXD/MNT in *Drosophila*) display significantly augmented growth and development accompanied by a partial restoration of the expression of dMyc activated growth genes [99]. Interestingly a similar type of rescue dynamic has been reported for other antagonistic pairs of transcription factors [100,101].

### Regulation through degradation

**Fig. 5:**
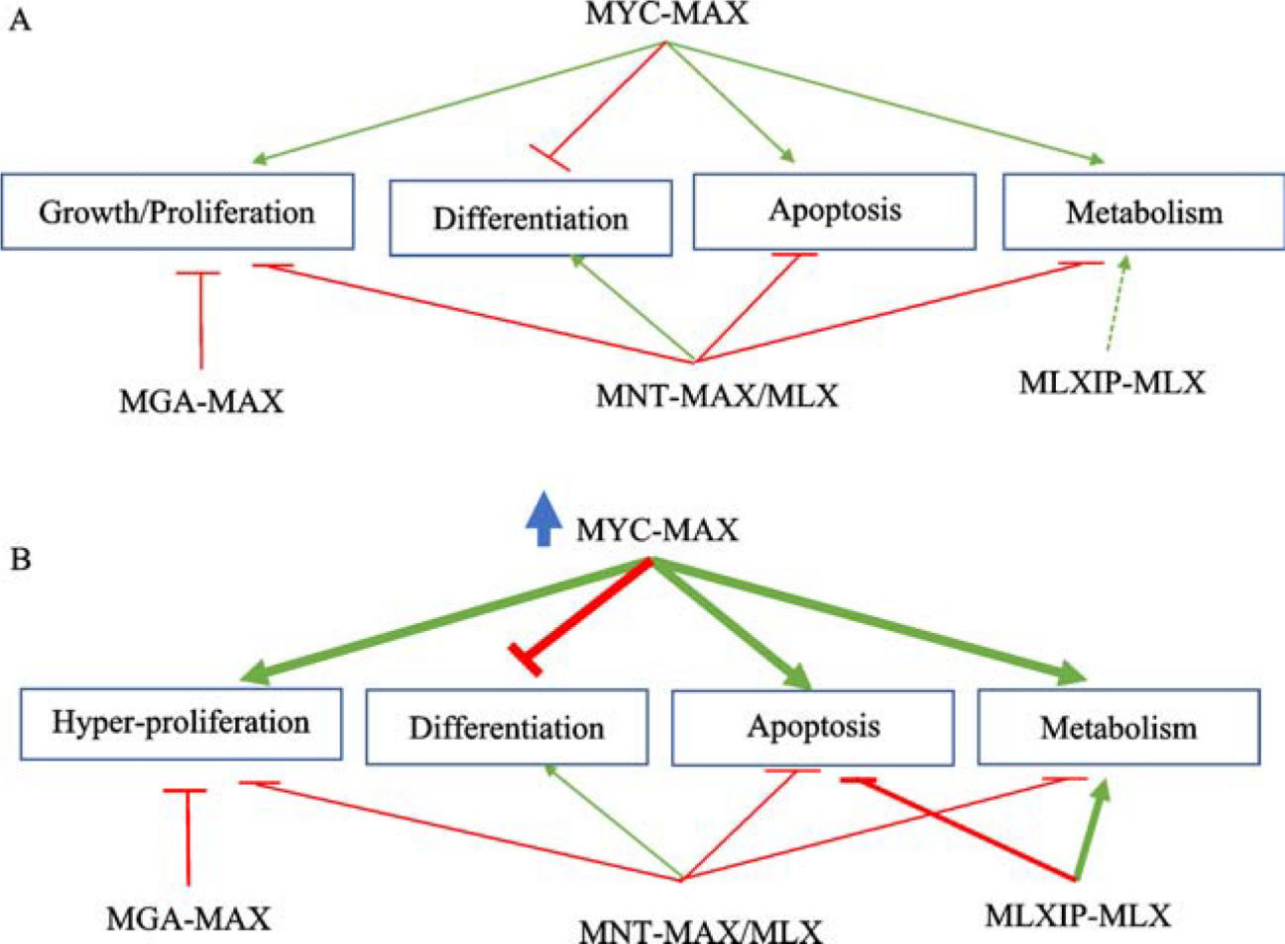
A hypothetical representation of two states of the MYC network and their impact on gene expression programs that influence growth, proliferation, differentiation, apoptosis and metabolism. (A) A balanced network in which gene expression is controlled through normal endogenous regulation of the MYC network. Transcriptional effects of MYC-MAX are balanced by MNT (heterodimerized with either MAX or MLX), MGA-MAX, and, under conditions of stress, by MLXIP-MLX or MLXIPL-MLX. (B) An unbalanced network due to deregulation of MYC expression. In this state MYC-MAX suppresses differentiation, reprograms metabolism, and triggers the apoptotic pathway. The suppressive effects of MGA-MAX and MNT-MAX/MLX on proliferation are overwhelmed by MYC-MAX which contributes to suppression of MYC-induced apoptosis. Increased nuclear accumulation of MLXIP-MLX (in response to deregulated MYC and/or metabolic stress) adjusts metabolic reprogramming by MYC-MAX and further reduces apoptosis. The effects on gene expression are presumed to occur through genomic binding and co-occupancy by network members. Green arrows, transcriptional activation; red arrows, transcriptional repression. Arrow width proportional to estimated transcriptional effect. Diagram adapted from [5].

Taken together, the evidence suggests that a regulated balance between MYC and MXD/MNT factors is important in maintaining growth homeostasis in response to environmental signals (Fig. 5). In the case of the MYC module, the signals leading to induction and maintenance of MYC expression can be generally classed as mitogenic and include a broad range of growth factors and cytokines including CSF-1, LIF, Wnt, Notch, Sonic Hedgehog, EGF, and IL2. While the MXD module is generally considered to be induced by growth arrest, as well as by developmental, and differentiation signals, the specific pathways leading to MXD induction are not well defined. Because the MYC family and MXD/MNT/MGA proteins all possess relatively short protein half-lives, regulation of their degradation is also an important aspect of setting the balance between these modules. MYC degradation is mediated by several ubiquitin ligases, one of which (FBXW7), requires a specific set of phosphorylations within a phosphodegron near the MYC N terminus (Fig. 3) [102]. As these phosphorylation events are stimulated by growth factor responsive signal transduction pathways such as RAS-MAPK and PI3K-AKT-GSK3β, it is evident that MYC degradation is responsive to environmental cues (for review see [103]). Interestingly, MXD1 has also been shown to be degraded subsequent to PI3K/AKT and MAPK mediated phosphorylation [104] suggesting that environmental signaling through these pathways may contribute to the balance between MYC and MXD arms of the network (see Fig. 5).

### MAX inactivation

A loss of network balance may also come into play in the case of the surprising findings that inactivating mutations or deletions of MAX are associated with tumorigenesis, particularly involving cells of neuroendocrine origin [71,85]. MAX inactivation would be expected to cancel MYC’s tumorigenic functions. However loss of MAX should also result in reduction or loss of MXD family, MNT and MGA binding. Although MXD1, MXD4, and MNT might still retain some activity by dimerization with MLX (see Fig. 1), the balance among network members upon MAX loss is likely to be seriously compromised, perhaps leading to aberrant activation of genes due to abrogation of MXD/MNT/MGA repression, similar to the effects observed upon double deletion of dMyc and dMnt in *Drosophila* as described above [99]. Another, not necessarily mutually exclusive, possibility is that MYC can retain certain critical functions in the absence of MAX, as suggested by MAX deletion experiments in *Drosophila* [105].

### Functional interactions between MYC-MAX and MLXIP-MLX

Given that deregulated MYC has been shown to stimulate aerobic glycolysis and to reprogram multiple aspects of metabolism [106] it is not entirely unexpected that important functional interactions exist between MYC-MAX and the MLXIP-MLX modules [11,21]. One aspect of this interaction was revealed in a recent study showing that, in a number of MYC-driven cancer cell lines (i.e., cells in which MYC is clearly deregulated and which depend on continued MYC expression for growth), the loss or suppression of MLXIP or MLX results in growth arrest and apoptosis, even though high MYC levels are maintained [107]. In a neuroblastoma cell line, MYC-MAX and MLXIP-MLX were found to cooperatively regulate transcription of a subset of genes involved in metabolism. These include genes encoding fatty acid synthase (FASN) and sterol CoA-desaturase (SCD), both rate limiting for fatty acid biosynthesis. Metabolomic and carbon tracing experiments demonstrated a significantly decreased contribution to the production of palmitate, an initial step in the fatty acid biosynthesis pathway. The resulting metabolic stress and cell death can be rescued by either restoring MLXIP expression, or by addition of the C18.1 monounsaturated fatty acid, oleate, supporting a critical role for lipid production in the survival of these tumor cells [107]. Importantly, cells in which MYC expression is under normal regulation are largely unaffected by MLXIP-MLX loss of function. This suggests that at least one aspect of MLXIP-MLX cooperativity with MYC-MAX is to modulate the expression of metabolic genes, such as FASN and SCD, in order to meet the increased metabolic demands imposed by MYC-MAX driven cell transformation. In this scenario, deregulated MYC, unlinked from its normal physiologic regulators, drives anabolic metabolism and proliferation regardless of the availability of extracellular factors that are normally limiting for cell growth. This imbalance is expected to result in stress, eventually leading to growth arrest and apoptosis. However the stress response is at least partially attenuated by MLXIP-MLX which remains responsive to nutrient availability and can be thought of as a transcriptional gatekeeper by adjusting gene expression in order to match metabolic demand [108] (Fig. 5).

### Involvement of MLXIP-MLX in the response to cellular stress

If MLXIP-MLX functions to suppress stress in MYC-driven cancers we might anticipate its involvement in other forms of stress response. In this regard it has been reported that MLX in *C. elegans* stimulates expression of autophagy genes subsequent to golgi disruption [109] and acts to inhibit TOR to promote longevity [110]. This is consistent with a report that MLX is responsive to, and suppresses, golgi stress in mammalian cell culture [110]. In addition, MLX null mice fail to regenerate muscle, compared to the rapid recovery of wildtype mice, following damage due to cardiotoxin treatment [111]. Furthermore, normal physiologic processes entailing high metabolic demand, such as T cell activation and spermatogenesis, are impaired in the absence of MLX (P.A.C. and B.W.F. manuscript in preparation). While MYC is known to be critical in both spermatogenesis [112] and T cell activation [30], it is unclear whether MLXIP-MLX are acting cooperatively with MYC-MAX in the manner described above for MYC-driven neuroblastoma and other tumor types [107]. Moreover in a triple negative breast cancer (TNBC) cell line, and in BRAF mutant melanomas, MYC and MLXIP appear to have opposing activities, at least with regards to inducing expression of the TXNIP target gene which encodes a negative regulator of glucose transport [113,114]. For example, in the highly glycolytic TNBC line, knockdown of MYC leads to MLXIP-mediated activation of TXNIP and decreased glucose uptake, indicating that MYC and MLXIP are acting antagonistically [113]. These diverse examples imply that the precise mechanisms underlying functional interactions between MYC-MAX and MLXIP-MLX are likely to depend on the specific biological context in which they occur and the unique bioenergetic or metabolic wiring present in those systems. Nonetheless, the available data support the overarching concept that these network interactions serve to modulate metabolism at the transcriptional level in order to balance nutrient supply and demand and influence cell fate [115].

## Genomic binding by MYC network heterodimers

The integrated functions of the network are dependent on the binding of heterodimeric complexes of network members to DNA (Fig. 1). Indeed, it is reasonable to surmise that the cooperative or antagonistic interactions among module heterodimers arises from direct interactions between these proteins at target gene loci. Genomic occupancies of many of these proteins have been mapped by the Encode project (www.encodeproject.org). However, as mentioned above, MYC-MAX binds to thousands of genes whose identity is dependent both on the nature of the cellular transcriptome and the abundance of MYC protein [40–42]. It is plausible to assume that binding by other network members will also be dependent on cellular context. Therefore, genomic occupancy studies of the MYC network will need to be carried out in biological settings in which network function is active and relevant and can be perturbed to reveal its critical functions.

A recent study employing *in vitro* selection for co-bound factors found that cooperative transcription factor binding to DNA is surprisingly common, estimating that ~25 000 distinct pairs of transcription factors may be associated with mammalian genomic DNA [116](for review see [117]). Cooperative binding is thought to contribute to the presence of dense clusters of transcription factors as observed primarily in nucleosome-free regions [118]. At the simplest level the dimerization of MAX with MYC or MXD proteins represents cooperative binding since these proteins do not recognize DNA as monomers nor form stable homodimers. However, an important question is whether heterodimers from the different arms of the MYC network interact cooperatively with DNA. Interestingly, structural studies indicate that MYC-MAX heterodimers could themselves dimerize to form heterotetramers in solution [1] and MLXIPL-MLX is thought to function as a dimer of dimers on the tandem ChoRE E-boxes in a cooperative manner [119]. Detailed analysis remains to be carried out on cooperative binding by different network heterodimers, but initial work supports the idea that MYCN-MAX and MLXIP-MLX can co-occupy the TXNIP promoter region and act to cooperatively augment TXNIP expression in MYC-driven neuroblastoma [107]. Cooperative binding is likely to account for the cooperative effects of MYC-MAX and MLXIP-MLX on expression of other metabolic genes and may account for antagonistic activity of MYC and MXD proteins (Fig. 5).

A detailed description of the network will involve mapping the genomic binding sites of all members of the three network modules as well as the effects of genetic and chemical perturbation of the network on genomic binding and expression of bound genes. In addition it is worth noting that the MYC network itself belongs to the larger super-family of bHLH transcription factors that recognize E-box DNA [120]. These include proteins such as CLOCK-BMAL and HIF among many others, whose functions impinge on MYC network activity [121,122]. Given the importance of the MYC network, we anticipate that the elucidation of its genomic binding, functional interactions, and its integration with other cellular transcriptional networks, will lead to deeper insights into normal cellular functions and provide new pathways and targets for cancer therapy.
